# Early mobilization and recovery in mechanically ventilated patients in the ICU: a bi-national, multi-centre, prospective cohort study

**DOI:** 10.1186/s13054-015-0765-4

**Published:** 2015-02-26

**Authors:** The TEAM Study Investigators

**Affiliations:** ANZIC-RC, Level 6, 99 Commercial Road, Prahran, VIC 3084 Australia

## Abstract

**Introduction:**

The aim of this study was to investigate current mobilization practice, strength at ICU discharge and functional recovery at 6 months among mechanically ventilated ICU patients.

**Method:**

This was a prospective, multi-centre, cohort study conducted in twelve ICUs in Australia and New Zealand. Patients were previously functionally independent and expected to be ventilated for >48 hours. We measured mobilization during invasive ventilation, sedation depth using the Richmond Agitation and Sedation Scale (RASS), co-interventions, duration of mechanical ventilation, ICU-acquired weakness (ICUAW) at ICU discharge, mortality at day 90, and 6-month functional recovery including return to work.

**Results:**

We studied 192 patients (mean age 58.1 ± 15.8 years; mean Acute Physiology and Chronic Health Evaluation (APACHE) (IQR) II score, 18.0 (14 to 24)). Mortality at day 90 was 26.6% (51/192). Over 1,351 study days, we collected information during 1,288 planned early mobilization episodes in patients on mechanical ventilation for the first 14 days or until extubation (whichever occurred first). We recorded the highest level of early mobilization. Despite the presence of dedicated physical therapy staff, no mobilization occurred in 1,079 (84%) of these episodes. Where mobilization occurred, the maximum levels of mobilization were exercises in bed (N = 94, 7%), standing at the bed side (N = 11, 0.9%) or walking (N = 26, 2%). On day three, all patients who were mobilized were mechanically ventilated via an endotracheal tube (N = 10), whereas by day five 50% of the patients mobilized were mechanically ventilated via a tracheostomy tube (N = 18).

In 94 of the 156 ICU survivors, strength was assessed at ICU discharge and 48 (52%) had ICU-acquired weakness (Medical Research Council Manual Muscle Test Sum Score (MRC-SS) score <48/60). The MRC-SS score was higher in those patients who mobilized while mechanically ventilated (50.0 ± 11.2 versus 42.0 ± 10.8, *P* = 0.003). Patients who survived to ICU discharge but who had died by day 90 had a mean MRC score of 28.9 ± 13.2 compared with 44.9 ± 11.4 for day-90 survivors (*P* <0.0001).

**Conclusions:**

Early mobilization of patients receiving mechanical ventilation was uncommon. More than 50% of patients discharged from the ICU had developed ICU-acquired weakness, which was associated with death between ICU discharge and day-90.

**Clinical trial registration:**

ClinicalTrials.gov NCT01674608. Registered 14 August 2012.

## Introduction

Globally, each year, millions of patients are discharged from hospital after surviving a critical illness. The consequences of critical illness and therapies administered in the ICU persist beyond hospital discharge and may contribute to poor post-ICU recovery [1,2]. Studies of long-term recovery after critical illness demonstrate that some patients experience profound and prolonged neuromuscular dysfunction [3,4]. Muscle weakness and wasting and nerve injury or damage resulting in ICU-acquired weakness (ICUAW) appears to start within the first few days of critical illness [5,6]. As such, interventions that reduce ICUAW and improve recovery after critical illness are of major importance to public health.

Recent point-prevalence studies have reported mobilisation practices across multiple ICUs in 1 day [7,8]; however, observational studies provide a more detailed opportunity to observe practice over a period of time. This includes time to initiation of mobilisation, frequency of mobilisation over a period of time and associations with outcomes. Observational studies have reported an incidence of ICUAW between 25 and 57%, depending on the ICU population being studied, and an association between ICUAW and increased duration of mechanical ventilation, increased length of stay in the ICU and hospital, poor functional recovery and reduced return to work among survivors [9,10]. From the results of propensity matched cohorts it is possible that ICUAW may have an effect on long-term survival [11], but this has relationship not been established from prospective data.

ICUAW is multifactorial, with myopathy, neuromyopathy and disuse atrophy all potential contributors, but immobility alone is known to result in loss of strength, muscular endurance and muscle bulk [5]. Early mobilisation, exercising patients while they are still receiving mechanical ventilation, is a candidate intervention to attenuate ICUAW and improve outcome [12,13].

The aims of this study were to report observed early mobilisation while on mechanical ventilation and to assess the relationship between occurrence of ICUAW and subsequent recovery.

## Methods

### Study design and patients

Each participating centre’s Human Research Ethics Committee approved this study with either person-responsible consent or opt-out consent at the time of post-discharge follow-up (please refer to the Acknowledgements). Opt-out consent was approved at most centres because this was an observational study with no change to standard care. We recruited patients from 12 ICUs in Australia and New Zealand, including six tertiary hospitals, four metropolitan hospitals and two rural hospitals. Between August 2012 and March 2013 each unit recruited up to a maximum of 25 patients. Patients were eligible for inclusion if they were independently able to mobilise prior to the current hospital admission (this included patients who used a walking stick or gait aid to mobilise, but not patients that needed assistance from another person or a machine such as a wheelchair), had been in the ICU <72 hours, had been receiving invasive ventilation for >24 hours and had expected to stay invasively ventilated for at least the next 48 hours. Patients were excluded if they had one or more of the following: age <18 years, proven or suspected neurological impairment, inability to communicate in English, cognitive impairment prior to the ICU admission, unstable fractures or any other injury that would require specific medical bed rest orders, an ICU admission for palliative care or proven or suspected primary myopathic or neurological process associated with prolonged weakness or ICU readmission.

### Study procedures

We conducted the study in collaboration with the Australia and New Zealand Intensive Care Research Centre and the study was endorsed by the Australia and New Zealand Intensive Care Society Clinical Trials Group. We designed an online data case report form. We trained site investigators and research coordinators in all study procedures.

### Measurements and data collection

Research coordinators at each participating site screened the patients for eligibility and sought consent as required. Research coordinators collected demographic data including age, gender and body weight, admission source, functional co-morbidities using the functional co-morbidities index [14], date and time of ICU and hospital admission, and date and time of first intubation at the time of enrolment into the study. We recorded the APACHE II score utilising data from the first 24 hours of admission to ICU and daily therapeutic interventions, including administration of mechanical ventilation, vasoactive agents and renal replacement therapy.

Research coordinators or site investigators collected daily data for the first 14 days of mechanical ventilation or until ICU discharge or death, whichever occurred first. Data were collected daily at 12:00 noon and included physiological information, Richmond Agitation and Sedation Scale score [15] and maximum level of mobilisation using the ICU mobility scale [16]. Data were also collected daily during mechanical ventilation about physiotherapy–patient interactions. Every patient was assessed by a physiotherapist to determine their ability to perform early mobilisation, as part of the standard care in each participating hospital. For patients who received early mobilisation, we collected data for all of the physiotherapy sessions on the day that the patient was seen regarding the duration of mobilisation activities, types of mobilisation activities and co-interventions (such as continuous renal replacement therapy or vasoactive agents). For patients who did not receive early mobilisation, we collected the reported barriers to mobilisation.

Early mobilisation was defined as any active exercise where the patients could assist with the activity using their own muscle strength and control that occurred while the patient was receiving invasive ventilation [13] and was scored using the ICU mobility scale [16]. This included the activities of rolling, bridging, sitting, standing and walking, and upper and lower limb flexion and extension, and did not preclude the patient receiving assistance from staff or equipment [17,18].

Serious adverse events were prospectively defined as a fall, unplanned extubation, cardiac arrest, loss of an invasively inserted line and new-onset atrial or ventricular tachyarrhythmia, and were recorded during mobilisation sessions. We defined adverse events that required a mobilisation session to be stopped prematurely *a priori*. These events were a decrease in mean arterial pressure <60 mmHg or a decrease in oxygen saturation <88% for more than 3 minutes and occurrence of a new oxygen requirement for a fraction of inspired oxygen >0.6.

Research coordinators collected ICU and hospital outcome data in all patients, including mobilisation data such as time to first mobilisation activity, time to first sit out of bed, stand and walk. We defined successful extubation as cessation of mechanical ventilation without reinitiation of ventilation within 24 hours.

In patients who were discharged from the ICU during business hours, physiotherapists assessed muscle strength using the Medical Research Council Manual Muscle Test Sum Score (MRC-SS) [19]. Full strength is a MRC-SS of 60/60 and ICUAW has been defined as a score <48/60 [20]. We recorded survival status at ICU and hospital discharge and day 90. We contacted patients by telephone at 6 months to establish their level of mobilisation using the ICU mobility scale [16], survival status, health-related quality of life using the EuroQoL standardised health outcome tool [21] and status relating to undertaking paid work.

### Statistical analysis

Normally distributed variables were compared using Student’s *t* test and reported as mean (standard deviation), while non-normally distributed variables were compared using Wilcoxon rank-sum tests and reported as median (interquartile range). Survival analysis between patients with and without ICUAW at ICU discharge was presented using a Kaplan–Meier curve with comparisons between groups performed using a log-rank test. Statistical analysis was performed using SAS version 9.3 (SAS Institute Inc., Cary, NC, USA). Two-sided *P* = 0.05 was considered statistically significant.

## Results

### Patient characteristics

**Figure 1:**
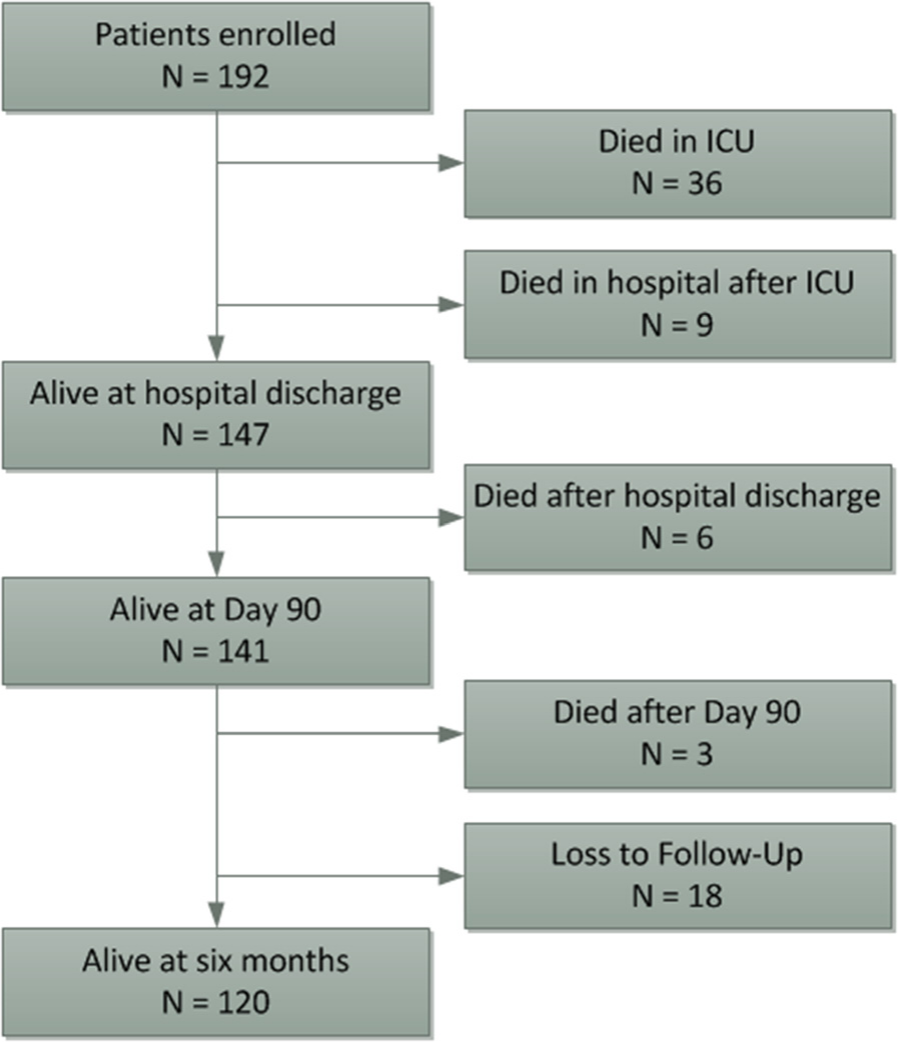
**Flow of patients through the study.**

A total of 192 patients met the inclusion criteria with no exclusions (Figure 1). There were 117 (61%) males and the overall mean age was 58.0 ± 15.8 years; 57 patients (30%) had no co-morbidities while overall there was a low functional co-morbidity index of 1 (interquartile range (IQR) 1 to 2) [22]. The mean Acute Physiology and Chronic Health Evaluation II score was 19.1 ± 7.6, which was higher than the overall Acute Physiology and Chronic Health Evaluation II score for the ICUs during this time period (mean 15.5 ± 5.6). Baseline patient characteristics are presented in Table 1. Patients were admitted to tertiary centres (*n* = 95, 49%), metropolitan centres (*n* = 9, 46%) and rural centres (*n* = 8, 4%).

**Table 1:** **Baseline data for all patients, patients with strength assessed at ICU discharge and patients without ICU-acquired weakness at ICU discharge**

**Baseline data**	**Total**	**Patients with strength assessed at ICU discharge**	**Patients without ICU-acquired weakness at ICU discharge**
	**(** ***n*** **= 192)**	**(** ***n*** **= 94)**	**(** ***n*** **= 46)**
Age (years)	58.0 ± 15.8	57.3 ± 15.5	57.1 ± 16.1
Male	117 (61%)	57 (61%)	29 (63%)
Previously walking independently	192 (100)	94 (100)	46 (100)
Weight (kg)	85.1 ± 25.2	82.5 ± 18.2	81.7 ± 16.4
APACHE II score	19.1 ± 7.6	19.5 ± 7.2	19.2 ± 6.9
Vasoactive drugs	127 (68%)	57 (62%)	29 (63%)
Time from ICU admission to enrolment (days)	2(1 to 2)	2 (1 to 2)	2 (1 to 2)
Functional co-morbidity index	1 (1 to 2)	1 (1 to 2)	1 (1 to 2)
Principal diagnoses	59 (31%)	28 (30%)	13 (28%)
Cardiac or cardiothoracic	40 (20%)	18 (19%)	10 (22%)
Respiratory	34 (18%)	16 (17%)	7 (15%)
Gastrointestinal	28 (15%)	13 (14%)	6 (13%)
Sepsis	31 (16%)	19 (20%)	10 (22%)
Other			

Data presented as mean ± standard deviation, number (percentage) or median (interquartile range). APACHE, Acute Physiology and Chronic Health Evaluation.

The use of vasopressors (*n* = 127, 66%) and deep sedation (*n* = 124, 64%) were common. The main reported barriers in patients who did not receive early mobilisation were intubation and sedation (Table 2). The median (IQR) duration of ICU length of stay was 11 (6 to 17) days. Nine patients (5%) were readmitted to the ICU after discharge. Overall ICU mortality was 36/192 (18.8%) and 90-day mortality was 26.6% (51/192) (Table 3). Of the 147 patients who survived to hospital discharge, 80 (54%) were discharged from hospital to home, 35 (24%) were discharged to another acute hospital and 32 (22%) were discharged to a rehabilitation centre.

**Table 2:** **Number of reported barriers to mobilisation in mechanically ventilated patients from days 1 to 7**

	**ETT**	**Sedation**	**Inotropes**	**Femoral line**	**Respiratory rate**	**PA catheter**	**Agitated**	**Weakness**
Day 1 (*n* = 192)	94	92	14	22	9	14	0	0
Day 2 (*n* = 181)	87	85	14	21	7	14	0	0
Day 3 (*n* = 161)	65	69	9	18	9	7	5	0
Day 4 (*n* = 147)	57	58	9	11	7	5	7	2
Day 5 (*n* = 131)	46	47	9	14	9	2	14	6
Day 6 (*n* = 108)	41	42	5	9	12	0	9	10
Day 7 (*n* = 101)	30	36	5	11	16	0	13	11

Each patient could have more than one barrier reported by ICU staff. ETT, endotracheal tube; PA, pulmonary artery.

**Table 3:** **ICU, hospital and survival outcomes of all patients**

**Study outcome**	
Time to first physiotherapy assessment (days)	2 (2 to 4)
Time to sit out of bed (days)	7 (4 to 10)
Time to first stand (days)	7 (4 to 11)
Time to walk (days)	9 (5 to 16)
ICU-acquired weakness^a^	48/94 (52%)
Ventilation days	8 (5 to 14)
ICU length of stay	11 (6 to 17)
Hospital length of stay	24 (16 to 42)
ICU mortality	36 (18.8%)
Hospital mortality	45 (23.4%)
90-day mortality	51 (26.6%)

Data presented as median (interquartile range) or number (percentage). ^a^ICU weakness measured at ICU discharge.

### Mobilisation activities

**Figure 2:**
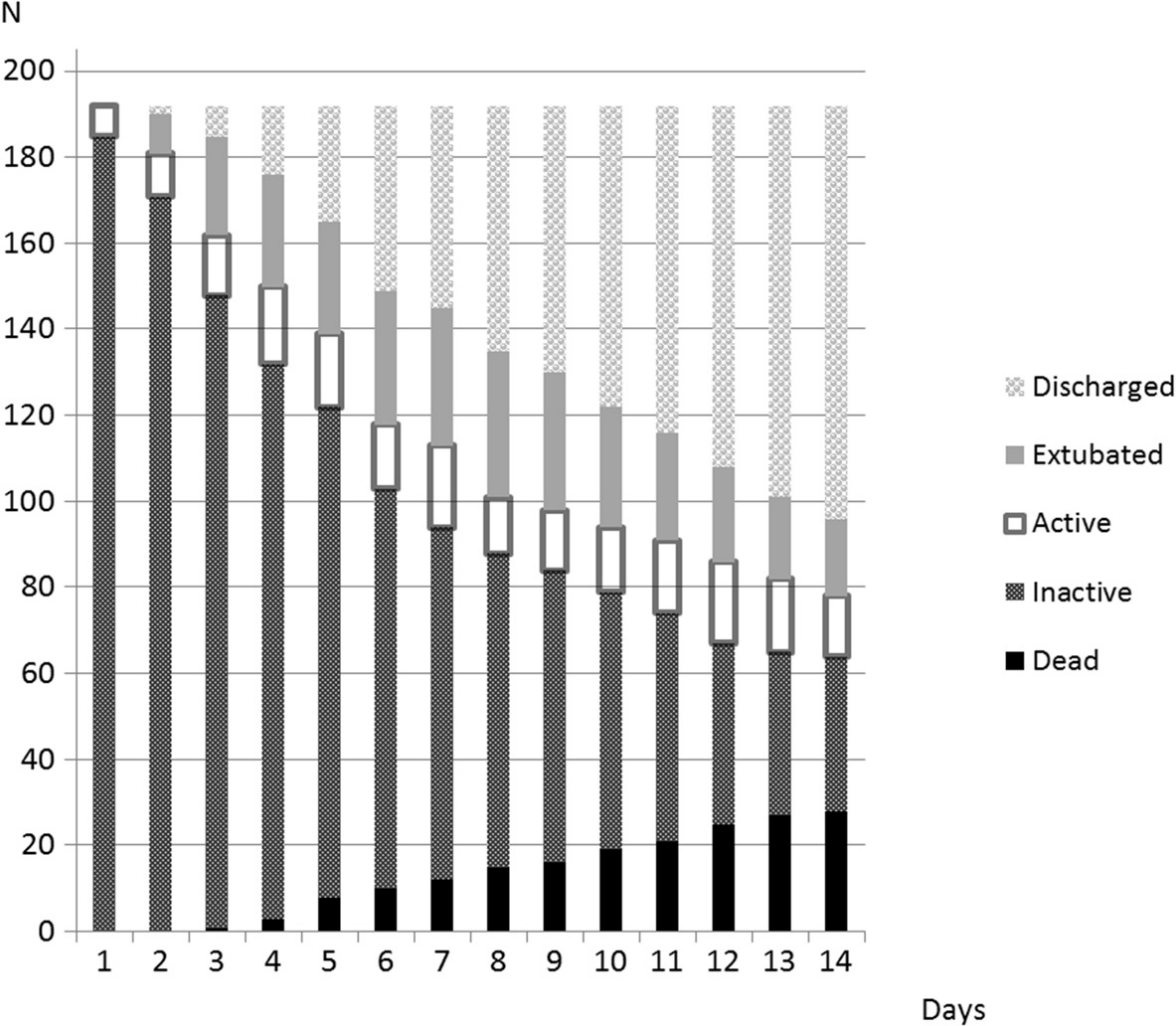
**Flow of included patients through the study from days 1 to 14.** Data for the number of patients invasively ventilated and mobilising (active), invasively ventilated and inactive, dead, extubated or discharged from the ICU.

Of our cohort, 122 (63.5%) patients did not receive early mobilisation. We collected information during 1,288 patient–physiotherapy interactions while patients were mechanically ventilated (Figure 2). No early mobilisation occurred in 1,079 (84%) of these episodes. The first physiotherapy sessions occurred early in the ICU stay (median 2 days from ICU admission, IQR 2 to 4 days) as the patients could be seen for respiratory physiotherapy or for early mobilisation.

**Figure 3:**
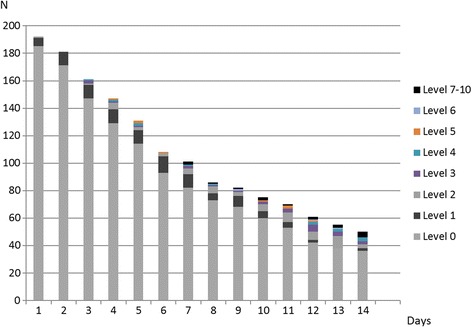
**Maximum level of activity in invasively ventilated patients for days 1 to 14.** Measured using the ICU mobility scale, where 0 = no activity, 1 = exercises in bed, 2 = passively moved to the chair, 4 = sitting on the edge of the bed, 5 = standing, 6 = transferring from bed to chair through standing, 7 = marching on the spot, 8 = walking with assistance of two people, 9 = walking with assistance of one person and 10 = walking independently.

Of the 70 patients (36.5%) who received early mobilisation during mechanical ventilation, the median (IQR) time from ICU admission to early mobilisation was 5 (3 to 8) days and the median (IQR) number of active mobilisation episodes per patient was 2 (1 to 4). There was no difference in any baseline variables between patients who were mobilised compared with patients who were not mobilised. There were 209 recorded episodes of early mobilisation. Among these episodes, the maximum levels of mobilisation were exercise in bed (*n* = 94, 45%), passively transferred to sitting (*n* = 52, 25%), sitting over the edge of the bed (dangling, *n* = 22, 11%), standing at the bedside (*n* = 11, 5%), transferring from bed to chair through standing (*n* = 4, 2%) or walking (*n* = 26, 12%) (Figure 3). One-quarter of these patients were mobilised by day 3 and one-third by day 4. No mechanically ventilated patients were walking before day 7. All patients who were mobilised out of bed and stood had a Richmond Agitation and Sedation Scale score of –1 to +1, while some patients were able to sit over the edge of the bed with a Richmond Agitation and Sedation Scale score of between –2 and +2.

There were no serious adverse events reported during mobilisation, although early cessation of mobilisation as a result of cardiovascular or respiratory instability occurred in 0.4% (six of the 1,288) physiotherapy–patient interactions. None of these required medical intervention.

### Muscle strength at ICU discharge

Physiotherapists measured MRC-SS in a subset of 94 (60%) of the 156 ICU survivors. The baseline demographics of these patients were similar to the entire cohort (Table 1). In the patients with MRC-SS measured at ICU discharge, the MRC-SS score (mean ± standard deviation) was 43.3 ± 12.5 and 49 (52%) of the patients had ICUAW (defined as MRC-SS <48/60) [23] at ICU discharge. There was no difference between patients with ICUAW and patients without ICUAW for age, Acute Physiology and Chronic Health Evaluation II score or functional co-morbidity index.

**Figure 4:**
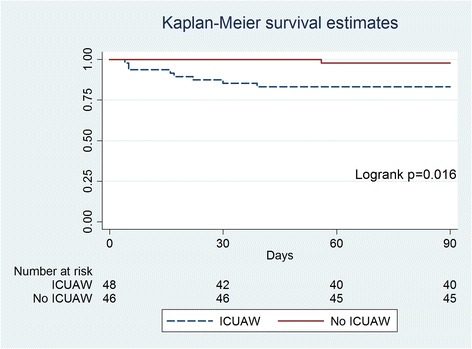
**Survival to day 90 in patients who survived to ICU discharge and were diagnosed with ICU-acquired weakness compared with patients without ICU-acquired weakness.** ICUAW, ICU-acquired weakness.

A higher MRC-SS score was associated with those patients who mobilised early while mechanically ventilated (50.0 ± 11.2 vs. 42.0 ± 10.8, *P* = 0.003) and those patients who were discharged home compared with all other patients discharged alive to other acute hospitals, rehabilitation or chronic care facilities (48.9 ± 10.3 vs. 37.8 ± 11.6, *P* <0.0001). The MRC-SS in patients who survived to day 90 was higher than those who survived to ICU discharge but who had died by day 90 (44.9 ± 11.4 vs. 28.9 ± 13.2, *P* <0.0001). Similarly, patients who survived to ICU discharge and were diagnosed with ICUAW by MRC-SS score demonstrated decreased survival to day 90 (Figure 4).

### Six-month outcomes

Of the entire cohort, 141 (73%) patients survived to day 90, and 120 (62%) patients survived to 6 months and responded to our telephone interview. Three patients died between day 90 and 6-month follow-up, while 18 patients (9%) were lost to follow-up. Of the 120 patients contacted at 6 months, 38% reported moderate–severe problems with usual care activities, 39% reported moderate–severe problems with anxiety and depression, 40% reported moderate–severe problems with mobility and 41% of patients reported moderate–severe problems with pain at 6 months based on the EuroQoL standardised health outcome tool (Table 4). Finally, 77 patients were working prior to ICU admission (Table 4). Only 29 (38%) of these patients had returned to work and only 25 (32%) had returned to work in their previous work role and hours.

**Table 4:** **Six-month outcomes**

**Study outcome at 6 months**	
Health-related QoL (*n* = 120)	
Problems with mobility	
No problems	72 (60%)
Some problems	46 (38%)
Confined to bed	2 (2%)
Problems with personal care	
No problems	97 (81%)
Some problems	19 (16%)
Unable to wash/dress	4 (3%)
Problems with usual activities	
No problems	68 (57%)
Some problems	42 (35%)
Confined to bed	4 (3%)
Pain/discomfort	
No pain/discomfort	71 (59%)
Some pain/discomfort	48 (40%)
Extreme pain/discomfort	1 (1%)
Anxiety/depression	
No anxiety/depression	73 (61%)
Moderate anxiety/depression	43 (36%)
Extreme anxiety/depression	4 (3%)
Best health (0 to 100)	69.5 ± 21.2
Return to work (*n* = 77)	29/77
Return to any work	29 (38%)
Return to previous work level	25/77 (32%)

Data presented as number (percentage) or mean ± standard deviation. QoL, quality of life measured with EuroQoL standardised health outcome tool [21].

## Discussion

### Key findings

This was the first detailed bi-national prospective, multicentre, observational cohort study of mobilisation practice in invasively ventilated patients. We found that no early mobilisation occurred in 84% of physiotherapy sessions in these patients. Importantly, this occurred in Australia and New Zealand where physiotherapists have been part of the ICU multidisciplinary team for decades and were available to treat patients early in the ICU stay, staffed at a median of one physiotherapist for every nine beds [24,25]. In a large subset of ICU survivors assessed for muscle weakness, more than 50% of patients were discharged from ICU with ICUAW. Increased muscle strength at ICU discharge was associated with early mobilisation during mechanical ventilation, discharge to home and increased survival at day 90.

### Relationship to previous studies

We defined early mobilisation as any early, active exercise during invasive ventilation, and it was not common in our cohort despite the fact that physiotherapy services were available to all ICU patients included in the study as part of standard ICU care. A snapshot of early mobilisation has been described previously in Australia and Germany in 1-day point-prevalence studies [7,8]. In the Australian point-prevalence study only 45% of the 498 patients included in the study were mechanically ventilated and no active mobilisation occurred out of bed in the patients who were mechanically ventilated. This was different to the German point-prevalence study the following year, in which all patients were mechanically ventilated (*n* = 783) and 24% of them mobilised out of bed, although only 8% of these had an endotracheal tube inserted. Importantly, these point-prevalence studies may include patients beyond 14 days of mechanical ventilation who have stabilised from the initial critical illness but have ongoing respiratory failure. In the current study, only 36.5% of mechanically ventilated patients received any active mobilisation and <10% of mobilisation episodes included activities out of bed, which may reflect the early time period of data collected (within 14 days of mechanical ventilation) and potential loss of data from patients with an ICU stay >2 weeks, although we know from previous work in our region that this is a very small percentage of the ICU patients [26]. The main reported barrier to mobilisation in our cohort was sedation, with nearly one-half of our cohort reported as too sedated for mobilisation on days 1 and 2 and >30% on days 3 and 4. This is different to the German study where only 15% of patients were reported as having sedation as the barrier to mobilisation. In a recent editorial accompanying the German point-prevalence study, Clemmer stated that, to successfully mobilise our patients, sedation, sleep and delirium monitoring must be routine and their barriers vigorously addressed [27]. In another study of barriers to mobilisation, the authors suggested that 47% of reported barriers to early mobilisation were potentially avoidable in a 4-week audit of 106 patients in the ICU [28].

In the current bi-national, multicentre cohort study, for the patients who received early mobilisation there was a median time of 5 days to early mobilisation and a median number of two early mobilisation sessions per patient. In one single-centre randomised controlled trial of ICU rehabilitation in Australia, early mobilisation was commenced 5 days after ICU admission and was not associated with improved outcome compared with standard care at ICU discharge, hospital discharge or 3, 6 or 12 months [29]. In this study, standard care included physical therapy in mechanically ventilated patients and the treatment group received additional physical therapy in ICU, on the ward and in an outpatient setting. Another study conducted in the United States has previously reported improved outcomes including time to liberation from ventilation and functional recovery, with patients receiving early mobilisation in the ICU compared with patients receiving standard care (no physiotherapy) [30]. It is not clear whether the discordant results between the Australian randomised controlled trial and the US randomised controlled trial are due to the timing of mobilisation activities, because the control arm received no physical or occupational therapy in the US study (a practice that is different to standard care in Australia, the United Kingdom and Europe), or other unknown factors.

In a recent systematic review and meta-analysis, Kayambu and co-authors found increased peripheral and respiratory muscle strength, reduced duration of ventilation, ICU and hospital length of stay and improved health-related quality of life in patients who received physical therapy in the ICU, including studies of interventions such as early mobilisation, cycle ergometery and electrical muscle stimulation [31]. It is not clear from this meta-analysis which intervention, timing of intervention or dosage is most effective to improve recovery in patients who survive the ICU stay. It is plausible that the first few days of invasive ventilation may be a key period for effectiveness trials, where early mobilisation interventions are most likely to have an impact on patient-centred outcomes if they can reduce ICUAW. The type of intervention, dosage and timing needs to be further investigated in large, randomised trials.

In our study, strength was assessed in a subset of patients at ICU discharge using the MRC-SS. Improved strength at ICU discharge was associated with discharge to home and survival to day 90. The rate of ICUAW was higher than previously reported in Australia, which may be reflective of the low occurrence of early mobilisation in our cohort [29]. Importantly, patients in the current study with higher strength scores at ICU discharge were more likely to survive to day 90. These results in a mixed medical and surgical population support previous findings in a surgical ICU population [11,32]. Other studies have had conflicting reports of an association between hand grip strength and mortality, which may indicate a lack of power to detect a difference [33,34]; however, a recent propensity-matched cohort study reported that ICUAW was associated with 12-month mortality [11]. Future studies will need to target interventions to reduce ICUAW to potentially improve long-term survival and recovery. In our cohort, less than one-third of patients had returned to their previous work at 6 months and 40% of patients were still reporting significant pain and reduced health-related quality of life.

Similar to previous studies [12], mobilisation was safe with no serious adverse events and only a small number of sessions ceased early as a result of physiological changes.

### Strengths and limitations

There were several strengths to this study. It was a multicentre, bi-national study of early mobilisation and the practices were consistent across the included hospitals. The data were collected prospectively. We made no assumptions for missing data at follow-up. There were several limitations of this study: we were unable to determine the total of number of patients screened because one study site did not record this information; we did not measure mobilisation episodes beyond 14 days or liberation from mechanical ventilation while the patients remained in the ICU, which may limit the data to patients in the critical phase of their illness and did not include the long-stay patients who might be mobilised after 14 days; and strength was assessed in a large subset of ICU survivors during business hours when trained physiotherapists were available to complete the MRC-SS, rather than in the entire cohort. While increased strength was associated with early mobilisation and survival at day 90, there may be unmeasured confounders that influence this result. This is an important area for future research. Finally, functional recovery measures were limited to return to work, ICU mobility scale score [16] and health-related quality of life.

This study has highlighted several areas for future research. The timing, dosage and mobilisation intervention needs to be further investigated and the association between early mobilisation, muscle strength and patient-centred outcomes should be tested in a multicentre randomised study.

### Implications of our findings

Early mobilisation in the ICU is not widely practised in Australia and New Zealand, despite the results of several small international studies that have demonstrated benefit [30,36-40]. If it is not happening in this region, where physiotherapists have been part of the ICU multidisciplinary team for decades, it is unlikely to be occurring elsewhere in the world other than in isolated ICUs with a strong culture of early mobilisation. The main barrier to mobilisation was intubation and sedation. This may suggest that unit culture rather than patient-related factors determined whether patients were mobilised. Sedation minimisation is an important component of the ability to mobilise patients early. In this case, the ICU culture should promote decreased sedation and early, active mobilisation as a priority.

## Conclusion

The majority of patients in Australia and New Zealand were not mobilised early while receiving mechanical ventilation in the ICU. The reported barriers to mobilisation were mainly intubation and sedation. Of our cohort of patients, who had few co-morbidities and were expected to recover, ICUAW at ICU discharge was common and 90-day mortality was high. Importantly, weakness was associated with mortality at day 90 among patients discharged from the ICU alive. Less than one-third of survivors had returned to their previous work at 6 months. This study provides information on current practice, key outcome rates and the rationale for the design of an interventional early mobilisation trial to test whether early mobilisation can improve patient-centred outcomes.

## Key messages

- Early mobilisation was not common in this cohort across multiple ICUs.
- The main barriers to mobilisation were intubation and sedation.
- Improved strength at ICU discharge was associated with early mobilisation and survival to day 90.

## Abbreviations

ICUAW: ICU-acquired weakness
IQR: interquartile range
MRC-SS: Medical Research Council Sum Score
